# From data to decision: a clinical pipeline for wearable AI in early cardiotoxicity detection

**DOI:** 10.1186/s40959-026-00506-x

**Published:** 2026-05-18

**Authors:** Ziqiang Zhou, Jinwen Wang

**Affiliations:** 1 Cardiovascular Center, Beijing Tongren Hospital, Capital Medical University, Dongjiaominxiang 1#, Dongcheng District, Beijing, 100730 China; 2 Beijing Institute of Heart, Lung and Blood Vessel Diseases, Beijing Anzhen Hospital, Capital Medical University, Chaoyang District, Beijing, 100029 China

**Keywords:** Cardio-oncology, Cardiotoxicity, Wearable sensors, Photoplethysmography, Artificial intelligence, Preventive cardiology

## Abstract

Chemotherapy-induced cardiotoxicity (CIC) is a leading cause of morbidity in cancer survivors, as conventional surveillance often detects cardiac dysfunction only after significant injury. This review moves beyond summarizing emerging technologies to focus on the end-to-end clinical pipeline—from sensor data to actionable decision-making—for creating a proactive “early-warning” system. We examine how continuous monitoring with wearable devices and artificial intelligence (AI) can detect subclinical CIC by analyzing digital biomarkers like heart rate variability. Proof-of-concept studies show AI can predict LVEF decline with moderate to high accuracy, though evidence is limited by small cohorts and lacks external validation. Implementing this wearable-AI pipeline could fill a critical surveillance gap and enable timely cardioprotective interventions. However, large-scale validation and building clinician trust through interpretable models are crucial before routine clinical adoption.

## Introduction

Chemotherapy and targeted cancer therapies have dramatically improved cancer survival rates, but this progress comes at the cost of cardiotoxicity—treatment-induced cardiac damage that can lead to heart failure, arrhythmias, or other cardiovascular complications [1]. Anthracycline chemotherapies (e.g., doxorubicin) and HER2-targeted agents like trastuzumab are particularly well-known for their cardiotoxic potential. Clinically, cancer therapy–related cardiac dysfunction (CTRCD) is often defined by a significant drop in left ventricular ejection fraction (LVEF) or the new onset of heart failure symptoms [2]. The incidence of cardiotoxicity varies significantly with the specific regimen and patient risk factors; for example, up to 27% of patients receiving trastuzumab may develop cardiotoxicity, a risk that can exceed 30% when combined with anthracyclines [3, 4]. The mechanisms of injury differ significantly: anthracyclines induce dose-dependent, irreversible myocardial damage primarily via topoisomerase IIβ inhibition and mitochondrial dysfunction, while trastuzumab’s cardiotoxicity is often reversible and linked to impaired myocardial repair and HER2 signaling disruption [5, 6]. As a growing number of patients achieve long-term remission, the burden of CTRCD has become a major clinical issue, with cardiovascular disease now a leading cause of non-cancer mortality among breast cancer survivors [7]. Early detection and prevention of cardiotoxicity are therefore a critical unmet need in modern oncology care.

### Clinical significance and the surveillance gap

Cancer therapy–related cardiac dysfunction (CTRCD) encompasses a spectrum ranging from subclinical myocardial injury to symptomatic heart failure, arrhythmias, myocarditis, hypertension, and thromboembolic events [1, 2]. Even mild treatment-related cardiac impairment can have outsized downstream consequences in oncology care: it may prompt dose reductions or treatment interruptions, constrain eligibility for subsequent lines of therapy, and increase long-term non-cancer morbidity in survivors [7]. Contemporary surveillance strategies are largely anchored to periodic imaging (left ventricular ejection fraction [LVEF], and increasingly global longitudinal strain) and intermittent biomarkers (e.g., troponin and natriuretic peptides) in selected risk groups [2]. While these approaches are essential, they are inherently episodic—often spaced weeks to months apart—creating a “blind interval” during which early physiologic drift can develop and remain undetected.

### Current cardiotoxicity detection methods and why early signals are missed

Current cardiotoxicity monitoring is optimized for identifying established dysfunction (e.g., LVEF decline) or overt myocardial injury (biomarker elevation), but it is less sensitive to early, dynamic perturbations that may precede these endpoints. Subclinical changes in autonomic balance, resting heart rate dynamics, rhythm instability, blood pressure trajectories, and functional capacity can emerge during or shortly after chemotherapy—sometimes before an echocardiographic abnormality is apparent [8]. These early signals are also frequently asymptomatic or non-specific (fatigue, reduced activity tolerance), and therefore under-reported by patients between appointments [9].

Continuous remote monitoring using wearable sensors represents a fundamental transition from periodic testing to real-time surveillance of a patient’s cardiovascular status. Wearable devices can track physiologic signals—heart rhythm, heart rate variability (HRV), blood pressure, and activity levels—continuously during a patient’s daily life, potentially revealing early abnormalities indicative of cardiotoxicity. Recent advances in artificial intelligence (AI) and machine learning are critical to this approach, enabling the detection of subtle patterns in these large-volume data streams that are not discernible by human clinicians. An AI-driven analysis pipeline can integrate multi-sensor data to generate an “early warning” of impending cardiac dysfunction. For instance, an algorithm trained on patterns like a sustained increase in resting heart rate and a decrease in HRV could prompt earlier diagnostic evaluation or prophylactic therapy [10]. By detecting cardiotoxic effects at a subclinical stage, this strategy aims to prevent irreversible cardiac damage and improve long-term outcomes. This narrative review provides a comprehensive overview of this emerging field, examining the wearable sensor platforms, data processing techniques, and AI models for predicting cardiotoxicity. We will also review current clinical evidence and discuss the challenges and future directions for clinical integration.

This review is organized to first describe the types of wearable sensors used for cardiac monitoring. We then outline how raw sensor data are processed into meaningful features for AI analysis. Following this, we discuss various AI and machine-learning models applied for cardiotoxicity prediction. We then review key clinical studies, summarize current challenges, and conclude with future directions for research and clinical practice. While a recent review has summarized the landscape of AI and smart devices in this field, our review provides a more in-depth focus on the clinical implementation pipeline, from raw sensor data to actionable decision support, and critically evaluates the specific machine learning architectures suited for this task [11].

## Literature search and review methodology

This article is a narrative review intended to synthesize the current state of wearable sensing and AI methods for early cardiotoxicity detection and to propose a practical, end-to-end clinical translation pipeline. It is not a systematic review and therefore does not follow PRISMA methodology or aim to produce a comprehensive quantitative effect estimate.

We performed a narrative search across PubMed/MEDLINE, Embase, Scopus, Web of Science, IEEE Xplore, and the ACM Digital Library, supplemented by Google Scholar, and ClinicalTrials.gov. The review covers the period from 2005 to 2025, prioritizing recent developments in AI/ML methods (2018–2025). Keywords included combinations of “cardiotoxicity,” “CTRCD,” “wearable sensors,” “photoplethysmography,” and “machine learning.” Manual screening of reference lists was also conducted to capture seminal works.

We prioritized English-language human studies and major guidelines focusing on cardiotoxicity surveillance or wearable AI methods. Given the nascent state of this field, we included a broad range of study designs, including prospective and retrospective cohorts, pilot feasibility studies, and methodological engineering reports, while excluding preclinical models. This narrative synthesis aims to describe the state of the art, identify gaps, and propose a clinically actionable pipeline, rather than to provide an exhaustive systematic census of all published reports.

## An end-to-end clinical pipeline for wearable-ai early cardiotoxicity detection

**Fig. 1:**
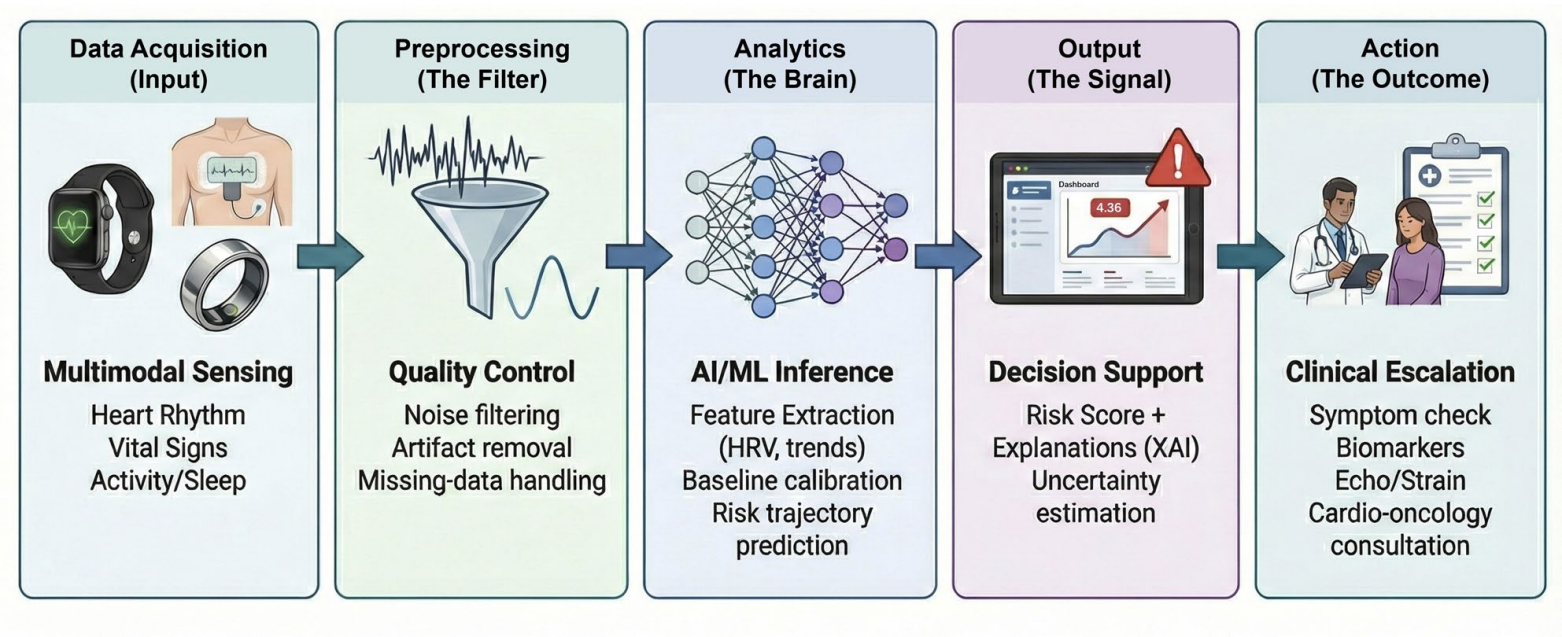
End-to-end wearable sensor–to–clinical decision pipeline for early cardiotoxicity detection. The workflow proceeds from data acquisition and quality control to AI/ML inference, generating interpretable decision-support outputs that trigger protocolized clinical escalation (e.g., symptom review, biomarkers, echocardiography/strain, cardio-oncology consultation) rather than autonomous diagnosis. Note: This figure depicts the operational data flow; patient selection and governance/safety considerations are described in the full clinical framework in the main text

A central barrier to real-world adoption of wearable-AI cardiotoxicity monitoring is that many reports focus on isolated components—either a device, an algorithm, or a single predictive endpoint—without specifying how the full system operates safely in clinical practice. Here we outline a pragmatic pipeline that links data acquisition to actionable decision-making (Fig. 1), with explicit attention to governance, safety, and feasibility.

### Step 1: patient selection, consent, and baseline calibration

Implementation should begin with clear eligibility criteria aligned to guideline-based risk (e.g., anthracycline exposure, HER2 therapy, immune checkpoint inhibitors, pre-existing cardiovascular disease, or multiple risk factors). Baseline calibration is essential because wearable signals are highly individualized. A pre-treatment baseline window (e.g., 7–14 days when feasible) can establish each patient’s resting heart rate distribution, nocturnal trends, HRV profile, rhythm irregularity burden, and activity/sleep patterns. This baseline-relative framing is often more robust than applying a single absolute threshold across heterogeneous patients.

### Step 2: device selection and data acquisition (fit-for-purpose monitoring)

Device selection should match the anticipated cardiotoxicity phenotype and the clinical question. For example, patch ECG may be preferred when rhythm/QT monitoring is critical; smartwatch PPG can support longitudinal trends in resting heart rate and HRV proxies; and multi-sensor systems can provide context (activity, sleep, respiratory rate) that improves interpretability. Data acquisition plans should specify wear time expectations, charging burden, skin considerations, and data transmission procedures, with patient-centered education to sustain adherence. Broad digital-health reviews emphasize that the technical success of wearable-AI systems depends as much on usability, connectivity, and privacy-preserving data flow as on the model architecture itself [12].

### Step 3: signal quality control, preprocessing, and feature engineering

Real-world wearable data are noisy and incomplete. The pipeline should incorporate automated signal quality indices, motion artifact handling, and missing-data strategies. Feature engineering should be clinically anchored (e.g., baseline-relative drift in nighttime HRV, sustained resting heart rate elevation, rhythm irregularity burden, or activity decline) and avoid overly complex, non-transportable feature sets that inflate in-sample performance but degrade external generalizability. Feature-selection reviews in adjacent medical domains highlight that aggressive or poorly validated feature selection can introduce instability and hidden bias [13], reinforcing the need for prespecified feature sets and external validation.

### Step 4: model inference with uncertainty, calibration, and interpretability

For clinical decision support, the model output should be more than a binary label. A deployable system should provide (i) a risk trajectory over time, (ii) uncertainty estimates or confidence intervals when feasible, (iii) calibration reporting (agreement between predicted and observed risk), and (iv) interpretable summaries that link the alert to measurable signal changes (e.g., “nocturnal RMSSD down 25% from baseline for 10 days; resting HR up 9 bpm from baseline; step count down 30%”). Explainable modeling and edge-enabled analytics have been proposed as strategies to improve transparency and feasibility in wearable deployments [14], particularly when bandwidth or privacy constraints limit continuous cloud transfer.

### Step 5: clinical decision support and escalation pathways (human-in-the-loop)

To prevent alert fatigue and unsafe automation, wearable-AI outputs should trigger protocolized escalation, not autonomous treatment. For example, a “moderate risk” alert may prompt symptom check-in and confirmatory biomarkers; a “high risk” alert may trigger expedited echocardiography/strain imaging or cardio-oncology consultation. The escalation pathway should specify responsibility (oncology vs. cardio-oncology vs. nurse-led monitoring team), documentation expectations, and timing. This governance layer is essential to transform model outputs into safe, reproducible care.

### Step 6: safety, ethics, and regulatory considerations

Wearable-AI cardiotoxicity monitoring raises distinct safety concerns: false positives may increase anxiety and unnecessary testing; false negatives may create false reassurance; and biased sensors/models may exacerbate disparities. Deployment should include (i) safety monitoring and auditing, (ii) subgroup performance evaluation (including skin tone and age when PPG is used), (iii) privacy/security safeguards, and (iv) clear patient communication regarding what the system can and cannot do. Smart-healthcare deployment frameworks emphasize that interoperability, cybersecurity, and governance are not ancillary—they are core determinants of whether AI monitoring can be adopted responsibly at scale [15].

### Advantages of an end-to-end pipeline (relative to isolated tools)

An integrated pipeline offers three practical advantages: (1) earlier detection of physiologic drift by leveraging baseline-relative continuous monitoring; (2) improved interpretability by contextualizing alerts with activity/sleep and by providing feature-level explanations; and (3) actionability through explicit escalation pathways that connect alerts to confirmatory testing and cardioprotective decision-making. These advantages are conceptual and require prospective evaluation, but they directly address why many promising models fail to translate into routine care.

### Wearable sensor technologies

The foundation of an early-warning system for cardiotoxicity rests upon wearable cardiac monitoring devices that collect physiological data in real-world settings. These technologies reduce reliance on in-hospital monitoring and can be categorized as follows:

1. Single-lead ECG devices

These devices capture a single-lead (and in some products, limited multi-lead such as three-lead) electrocardiogram (ECG) and are available in several form factors.

Patch ECG monitors (e.g., Zio^®^ Patch) are adhesive devices worn on the chest for continuous recording over 7–14 days. They are particularly effective for quantifying arrhythmia burden (e.g., atrial fibrillation/flutter, ectopy), pauses, and conduction abnormalities, and for longitudinal assessment of repolarization metrics such as QT interval trends when signal quality permits [16]. Importantly, patch ECG systems are not designed or validated as ischemia-detection tools in the manner of diagnostic 12-lead ECGs; therefore, in cardio-oncology they should be framed primarily as rhythm/conduction and repolarization monitors rather than ischemia screens. In the context of cardiotoxicity, increases in ectopy burden, new atrial arrhythmias, and dynamic QT prolongation (particularly in patients receiving QT-prolonging agents) may serve as actionable early markers that trigger confirmatory evaluation.

Handheld single-lead ECG devices (e.g., KardiaMobile^®^) allow patients to record a Lead I ECG on-demand. While not continuous, their portability facilitates frequent home-based rhythm surveillance. Studies have validated that such devices can accurately detect atrial fibrillation with high sensitivity and specificity when paired with appropriate algorithms [17]. Newer smartwatches now also incorporate on-demand ECG capabilities, blurring the lines between dedicated medical devices and consumer wearables.

2. PPG-based wearables (Photoplethysmography)

Photoplethysmography (PPG) is an optical technique used by nearly all smartwatches and fitness bands (e.g., Apple Watch^®^, Fitbit^®^) to measure pulse rate from the wrist. By detecting blood volume changes in capillaries, these devices provide continuous heart rate data. Their widespread use and 24/7 wear time yield a rich data stream for analysis. Although PPG is not an electrical measure, algorithms have been validated to screen for atrial fibrillation by detecting irregular pulse patterns [18]. Beyond heart rate, advanced PPG analysis can estimate heart rate variability (HRV) and even provide insights into vascular tone. For cardiotoxicity monitoring, these devices can flag abnormal trends, such as a persistent rise in resting heart rate or a decline in HRV, which may indicate developing cardiac dysfunction or autonomic shifts [10]. While PPG signals are susceptible to motion artifact, their ubiquity and continuous nature make them highly valuable for long-term, passive monitoring.

3. Multi-sensor platforms

Combining multiple sensors provides a more comprehensive assessment of cardiovascular status. Wearable vests or chest straps can integrate ECG, accelerometers, and thoracic impedance to simultaneously monitor heart rhythm, respiration, and activity. Other systems pair a wearable ECG with a separate blood pressure monitor or integrate data from multiple devices, such as a smartwatch and a smart scale. The integration of multi-sensor data helps differentiate true cardiac-related changes from other causes. For instance, an AI algorithm can correlate a heart rate spike with activity data to determine if it is a normal physiological response or a potential sign of an arrhythmia at rest. While combining signals from different devices can improve predictive accuracy [19], it requires robust software and cloud platforms for data aggregation and analysis, as illustrated in the overall technological pipeline (Fig. 1).

4. Regulatory status and patient acceptance

The regulatory status of wearables varies, ranging from consumer wellness gadgets to FDA-cleared medical devices. Several single-lead ECG devices and blood pressure wearables have received FDA clearance for arrhythmia detection or blood pressure measurement, respectively. This validation is crucial for clinician confidence. However, most devices are not specifically approved for “predicting cardiotoxicity,” a claim that will require further clinical evidence.

Patient acceptance in oncology has been generally positive in pilot studies, with many patients appreciating the proactive monitoring [20, 21]. Practical barriers include the need for device charging, skin irritation from adhesives, and data privacy concerns. To ensure adherence, devices must be user-friendly, and data transmission should be automated. Critically, AI-driven alert systems must be designed to minimize false alarms, which can cause patient anxiety and clinician alert fatigue. Early trials suggest that with good algorithm design, alert specificity can be optimized to an acceptable level for both patients and clinicians [22].

A comparative summary of these wearable device categories, including their strengths and limitations in cardio-oncology, is provided in Table 1.

**Table 1:** Wearable device categories for cardio-oncology monitoring: signals, strengths, and limitations

Wearable class	Primary signals	Typical monitoring pattern	Strengths (advantages)	Limitations (disadvantages)	Best-fit cardio-oncology use cases
Patch ECG (1–3 lead)	Continuous ECG (rhythm/conduction; QT trends if signal quality permits)	Continuous for 7–14 days	High-fidelity rhythm burden quantification; passive capture; strong for AF/ectopy/brady/pauses	Not validated for ischemia detection; skin irritation; finite wear window; data volume/QC needs	Arrhythmia surveillance; QT prolongation trend monitoring; suspected myocarditis/conduction changes (adjunct)
Smartwatch (PPG ± spot ECG)	PPG HR; HRV proxies; activity; sleep; sometimes single-lead spot ECG	Near-continuous PPG + intermittent ECG	High adherence/acceptability; multimodal context (activity/sleep); scalable remote monitoring	PPG artifact (motion/skin tone); HRV variability across devices; ECG is intermittent; “black-box” vendor algorithms	Detect physiologic drift (resting HR↑, HRV↓); contextualize symptoms; longitudinal recovery monitoring
Chest-strap ECG	ECG-derived HR/HRV; exercise HR dynamics	Intermittent/continuous during wear	Better HR/HRV fidelity than wrist PPG during activity; stable during exercise	Less comfortable; adherence may be lower; limited passive wear	Detailed HRV/chronotropic response assessment; rehab/return-to-activity monitoring
Ring-based PPG	HR; HRV proxies; sleep staging; temperature trends	Near-continuous with high wearability	Excellent overnight adherence; strong for nocturnal HR/HRV trends; low burden	PPG limitations remain; limited rhythm detail; device-specific algorithms	Nocturnal physiologic drift detection; recovery and sleep disruption tracking
Cuffless BP wearables (where available) + home cuff BP	BP estimates or cuff-based BP; sometimes HR/PPG context	Intermittent (daily/weekly)	Enables hypertension surveillance (TKI/VEGF inhibitors); actionable with clear targets	Cuffless accuracy varies; calibration required; adherence needed; confounding (stress/pain)	Hypertension early detection and management; longitudinal BP burden during therapy
Multi-sensor adhesive patches (ECG + accel + respiration/temp)	ECG; activity; respiration rate; temperature; posture	Continuous days–weeks	Rich context for false-alert reduction; better physiologic phenotyping	Skin issues; cost/logistics; integration complexity	Early decompensation detection (HR drift + activity decline + RR changes); acute toxicity monitoring feasibility studies
Smartphone-only (camera PPG / symptom apps)	PPG HR (intermittent); PROs/symptoms	Intermittent	Low barrier entry; easy PRO integration	Intermittent, higher missingness; signal variability	Symptom-triggered checks; PRO-driven escalation adjunct

Common wearable classes relevant to cardio-oncology monitoring with practical advantages/limitations. Selection should be “fit-for-purpose” (arrhythmia/QT vs. physiologic drift vs. BP/functional decline) and aligned with workflow capacity and patient burden

*Abbreviations: AF* atrial fibrillation, *AI* artificial intelligence, *BP* blood pressure, *ECG* electrocardiogram, *HR* heart rate, *HRV* heart rate variability, *PPG* photoplethysmography, *PROs* patient-reported outcomes, *QT* QT interval, *QC* quality control, *RR* respiratory rate, *TKI* tyrosine kinase inhibitor, *VEGF* vascular endothelial growth factor

### Data processing & feature extraction

Raw data from wearable sensors require significant processing to remove noise and extract reliable features for AI models. This process is essential for transforming noisy, real-world data into clinically meaningful indicators of cardiac function. The key categories of these extracted features and their clinical relevance are summarized in Table 2.

**Table 2:** Key features extracted from wearable sensor data for cardiotoxicity monitoring

Feature Category	Examples	Relevance to Cardiotoxicity	Notes / Limitations
Heart Rate & HRV (Time-Domain)	Resting HR; SDNN, RMSSD	Elevated resting HR and reduced HRV may indicate autonomic dysfunction and early cardiac stress [23, 24].	Non-specific; can be affected by stress, caffeine, etc. Requires baseline comparison.
HRV (Frequency-Domain)	LF/HF ratio	Increased sympathetic dominance (high LF/HF ratio) may reflect SNS overactivity preceding CTRCD [25].	Requires sufficient continuous data; influenced by breathing rate.
ECG Morphology	QT interval, ST changes, arrhythmia count	QT prolongation or frequent ectopic beats may be early indicators of cardiomyocyte stress [16].	Single-lead ECG may have limitations in accurately measuring all intervals compared to 12-lead.
PPG Pulse Waveform	Pulse amplitude, augmentation index	Changes in waveform shape can suggest reduced stroke volume or increased arterial stiffness [26].	Can be altered by user movement and peripheral vasoconstriction.
Activity & Sleep Metrics	Daily step count, sleep duration/quality	A decline in activity or poor sleep may reflect fatigue or reduced exercise tolerance from emerging heart failure [27].	Confounded by non-cardiac factors (e.g., pain, depression). Best used in combination with physiological metrics.

This table outlines key feature categories derived from wearables. Multimodal approaches that integrate several feature types are expected to yield the most robust predictive performance

*Abbreviations: CTRCD* cancer therapy–related cardiac dysfunction; *ECG* electrocardiogram, *HF* high-frequency, *HR* heart rate, *HRV* heart rate variability, *LF* low-frequency, *PPG* photoplethysmography, *RMSSD* root mean square of successive differences between normal heartbeats, *SDNN* standard deviation of all normal-to-normal intervals, *SNS* sympathetic nervous system

1. Signal Quality Control and Artifact Removal

The first step is to filter noise and remove artifacts caused by motion or poor sensor contact. For ECG, band-pass or adaptive filters are used to clean the signal. For PPG, techniques like wavelet denoising help identify true pulse peaks. Onboard algorithms often provide a signal quality index, and data segments below a certain quality threshold are typically discarded. This step is critical to prevent misinterpreting artifacts as pathological signals, ensuring the reliability of subsequent feature extraction.

2. Heart Rate Variability (HRV) and Related Metrics

HRV, the beat-to-beat variation in heart rate, reflects autonomic nervous system dynamics and has emerged as a promising biomarker for early cardiotoxicity. It can be derived from ECG or PPG recordings. Reductions in overall HRV or shifts in autonomic balance (i.e., sympathetic/parasympathetic tone ratios) may indicate early cardiac stress or subclinical LV dysfunction [23]. Time-domain metrics such as SDNN and RMSSD, and frequency-domain metrics like low-frequency (LF) and high-frequency (HF) power, are commonly used. Studies have shown that parasympathetic HRV indices often decline shortly after chemotherapy [28]. While limited studies have directly linked HRV to anthracycline-induced cardiotoxicity, neurohormonal changes and biomarker elevation have shown potential for early detection in prospective trials. In the PRADA study, circulating biomarkers outperformed baseline LVEF in predicting cardiac dysfunction during anthracycline therapy [24]. HRV metrics, including deceleration capacity, may provide additional sensitivity and can be calculated over sliding windows to monitor longitudinal trends throughout treatment.

3. PPG Morphological and Perfusion Markers

Beyond heart rate, the PPG waveform’s morphology contains valuable information about vascular health. Features such as pulse wave amplitude, shape, and timing can reflect changes in vascular compliance and peripheral perfusion. For instance, reduced pulse wave amplitude may indicate diminished stroke volume, while changes in the waveform shape can suggest increased arterial stiffness, a known side effect of some cancer therapies [26]. AI models can incorporate dozens of these PPG-derived features to detect patterns consistent with declining cardiac function or endothelial dysfunction.

4. Behavioral and Physical Activity Correlates

Wearables also track physical activity, which provides crucial context for interpreting physiological data and can serve as an independent marker of health. A key derived feature is resting heart rate, and an upward trend in this metric over weeks of chemotherapy has been associated with incipient heart failure [27]. Similarly, a decline in daily step count or overall activity level can indicate fatigue or exercise intolerance, which may be early signs of cardiac dysfunction. Data processing pipelines typically align physiological data with accelerometer data to classify periods as rest, activity, or sleep. This allows for state-specific analysis (e.g., calculating nighttime HRV) and helps ensure that observed physiological changes are not simply due to variations in activity levels. Machine learning models often integrate these behavioral features with physiological signals to improve the robustness of cardiotoxicity prediction [22].

### AI & machine-learning models

Raw and derived features from wearables become inputs for AI and machine-learning models that learn to recognize patterns associated with cardiotoxicity. Various modeling approaches have been explored, each with distinct advantages and challenges. Here we review the primary types of AI models applied or applicable in this domain.

1. Recurrent Neural Networks (RNNs) and LSTMs

Recurrent neural networks (RNNs), and their advanced variants like Long Short-Term Memory (LSTM) networks, are specifically designed to ingest time-series signals, such as daily heart rate profiles or continuous ECG data. Their key strength is the ability to capture temporal dependencies—effectively “remembering” what happened earlier in a sequence when interpreting later events. For example, an LSTM can be trained on daily HRV feature values during chemotherapy to predict a future LVEF drop. It can learn that a subtle HRV decrease sustained over a week, combined with a rise in blood pressure, is an ominous combination, whereas a momentary HRV dip that recovers is likely benign.

Proof-of-concept studies have demonstrated the feasibility of using LSTMs on wearable telemetry to detect patterns in heart rate and activity data that precede biomarker elevation [29]. While RNNs have proven utility in other patient monitoring tasks like arrhythmia detection, their specific application to cardiotoxicity is nascent. Key limitations include the requirement for large training datasets to prevent overfitting over months of monitoring, and the challenge of capturing very long-term dependencies in the data. Despite these hurdles, their success in analyzing sequential data makes them a natural choice for processing chronological health data to generate a real-time cardiac risk trajectory.

2. Transformer-Based Models for Time-Series

Transformers, adapted from natural language processing, are increasingly applied to medical time-series due to their strength in modeling long-range dependencies. The model’s “self-attention” mechanism can analyze an entire timeline of a patient’s sensor data—spanning multiple chemotherapy cycles—to learn which periods are most predictive of later cardiotoxicity. For instance, a Transformer could identify that a transient HRV drop during an early cycle is a strong harbinger of an eventual LVEF decline months later, assigning high importance to that early event when generating a risk prediction.

**Fig. 2:**
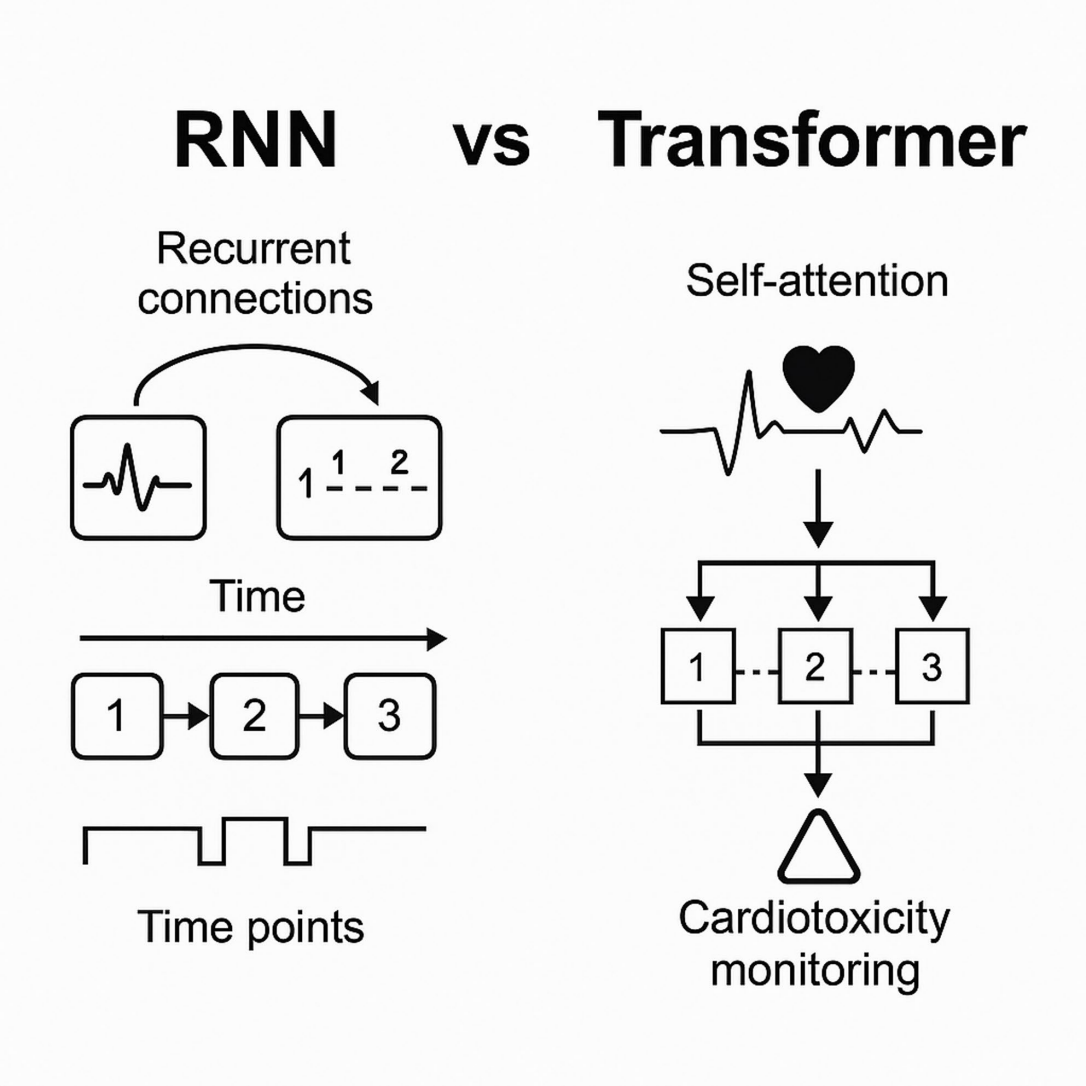
Comparison of RNN vs. Transformer architecture for time-series analysis in cardiotoxicity monitoring. RNNs (e.g., LSTM) process sequential data stepwise, capturing short-term dependencies, while Transformers use self-attention to capture long-range relationships between all time points, potentially identifying complex temporal patterns in a patient’s longitudinal data

Transformers handle longer sequences more effectively than RNNs, making them powerful for capturing trends over months as cardiotoxic effects gradually accumulate. This approach is analogous to using Transformers to forecast heart failure decompensation from remote monitoring data [30]. However, their main drawbacks are the need for very large training datasets and significant computational power, often necessitating data sharing across institutions. Current research is exploring hybrid models, for example combining Transformers with convolutional neural networks (CNNs) to analyze ECG data for cardiotoxicity prediction [31]. While still an emerging area, these architectures hold significant promise for improving prediction accuracy and interpretability. The architectural differences between sequential models are illustrated in Fig. 2.

3. Ensemble Methods (Random Forests, XGBoost, etc.)

Ensemble machine-learning methods, such as Random Forests (RF) and Gradient Boosted Trees (e.g., XGBoost), excel at analyzing structured, tabular data, making them well-suited for aggregated wearable sensor features and moderate-sized datasets. In this approach, a patient’s time-series data is summarized into key features (e.g., baseline HRV, average resting HR), which a classifier then uses to predict cardiotoxicity. For example, a study by Chang et al. used this methodology with clinical and imaging features from breast cancer patients, achieving a modest AUC of ~ 0.66 for predicting cardiotoxicity with an ensemble model [32]. This methodology is directly applicable to wearable data. Models like XGBoost can handle heterogeneous features and provide interpretable feature-importance rankings. A random forest, for example, might generate clinically intuitive rules by identifying key predictors such as a significant decrease in nighttime RMSSD. A major advantage is that ensemble methods are less data-hungry than deep learning, which is crucial given the small cohorts in many cardio-oncology studies. Their main limitation is the loss of temporal granularity by requiring a summary of the time-series into fixed features. Nevertheless, they have proven effective in related domains like predicting heart failure hospitalization [33] and are often the most practical and powerful choice for current datasets [34].

4. Unsupervised Anomaly Detection (Autoencoders, Clustering)

An alternative to supervised prediction is unsupervised anomaly detection, which models a patient’s normal physiological pattern to detect meaningful deviations. This approach is attractive because it does not require a large labeled dataset, allowing each patient to serve as their own control. For instance, autoencoders can be trained on a patient’s initial sensor data to establish a healthy baseline; a subsequent increase in the model’s reconstruction error can then flag a potential anomaly [35]. Clustering algorithms can similarly categorize data into “normal” versus “outlier” groups.

Unsupervised methods can also be applied across patients, for instance by clustering trajectories to identify if those who experienced cardiotoxicity form a distinct group. Autoencoder models have been explored on continuous ECG streams to detect subtle changes in waveform morphology or HR patterns that are not obvious [36]. The primary challenge with anomaly detection is tuning sensitivity versus specificity to avoid excessive false alarms from non-cardiac events. While hybrid approaches are being explored, the main benefit is the potential to discover novel, patient-specific patterns not in a pre-labeled training set, which is useful given the limited cases available for supervised training [37].

5. Model Interpretability and Clinical Integration

For AI models to gain acceptance in clinical cardio-oncology, interpretability is paramount. Clinicians are more likely to trust and adopt an AI system if it provides understandable reasoning for its predictions, a concept known as “explainable AI” (XAI). Several techniques are employed to achieve this:

- Feature importance and SHAP values: For ensemble models and some neural networks, SHAP (Shapley Additive Explanations) values can quantify how each input feature contributed to a particular prediction [38]. For example, an AI system might produce a high risk score for a patient and indicate that a decreasing HRV contributed + 0.15 to the score, while frequent ventricular premature beats contributed + 0.10. This aligns the model’s output with clinical reasoning.
- Attention weight visualization: In models like Transformers, attention weights can be visualized to show which time periods or sensor channels the model focused on when making a prediction. An attention plot might highlight that the model paid extra attention to the week following the third chemo cycle, suggesting something in that period was critical and prompting clinicians to review it more closely.
- Saliency and Grad-CAM for signals: Adapted from imaging, saliency mapping can be applied to 1-D signals like ECGs. One study used an AI to predict cardiotoxicity from 12-lead ECGs and was able to highlight subtle T-wave changes that the model found predictive, potentially revealing new ECG biomarkers [31].
- Human-AI interaction design: Integrating AI outputs into clinical workflows requires intuitive dashboards and alerts. A prototype system, “CardioAI,” demonstrated this by generating a risk score accompanied by a textual summary of key changes in the patient’s data and a chart highlighting out-of-range readings [22]. Clinicians in an evaluation study appreciated these explanations, which increased their confidence in the AI’s assessment.

Nevertheless, a fundamental challenge for current XAI methods is bridging the semantic gap between a model’s statistical patterns and clinically actionable pathophysiological insight. Techniques like SHAP and attention maps can reveal what features the model deemed important—for example, that a sharp decline in the RMSSD of HRV was highly predictive. However, they cannot inherently explain why this occurs from a biological standpoint; that is, they do not explicitly link the diminished RMSSD to the underlying mechanism of chemotherapy-induced autonomic nervous system dysregulation. This disconnect between statistical correlation and mechanistic reasoning remains a critical barrier to fostering deep clinical trust. The next frontier for XAI is therefore to evolve from mere feature attribution toward semantic interpretability, where model outputs are linked to established clinical knowledge graphs and pathophysiological pathways. Such an advancement is essential to transform the AI from a black-box oracle into a true collaborative partner in clinical reasoning.

Interpretability bridges the gap between complex algorithms and clinical judgment. It must be balanced with accuracy, as highly complex models may capture subtle signals that simpler, more interpretable models cannot. The goal is to augment these models with post-hoc explanation tools. In summary, while various AI strategies are being applied to early cardiotoxicity detection, the optimal solution may involve a combination of models. Regardless of the approach, building clinician trust through interpretable outputs is crucial for successful implementation.

### Clinical application studies

Early research efforts applying wearable sensors and AI in cardio-oncology have yielded promising, though preliminary, results. In this section, we review representative clinical studies across different chemotherapy agents and patient populations. We highlight study designs, sample sizes, types of data collected, AI methods used, and key performance outcomes for detecting cardiotoxic events. A summary of these representative studies is provided in Table 3.

**Table 3:** Representative clinical evidence for AI-enabled early cardiotoxicity detection

Study (Year)	Population / Therapy (Design; *N*)	Data modality (Wearable vs. clinical)	AI / analytics approach	Endpoint definition	Performance (as reported)	Clinical utility (advantages)	Key limitations / validation status (disadvantages)
Chang et al., 2022 [32]	Breast cancer; anthracycline (prospective; *N* = 130)	Clinical longitudinal data + serial echo/biomarkers (non-wearable)	Multiple ML classifiers incl. MLP (ensemble comparison)	CTRCD defined by LVEF drop and/or HF symptoms	AUC ≈ 0.66; Sens ~ 86%; Spec ~ 53%	High-sensitivity triage: can “cast a wide net” early to prioritize intensified surveillance and cardioprotective consideration	Modest discrimination; low specificity (false alerts); limited cohort/event size; no wearable data; external validation not established
Jacobs et al., 2024 [39]	Breast cancer post-anthracycline (*N* = 989)	Standard 12-lead ECG (clinical, not wearable)	Deep learning ECG model (CNN-style)	New abnormal LVEF (< 50%)	AUC ≈ 0.93	Scalable screening for asymptomatic LV dysfunction using widely obtainable ECGs; supports concept that subtle ECG features can precede LVEF decline	Primarily post-treatment screening; translation to single-lead/patch ECG not guaranteed; external validation/workflow impact not fully established
Yagi et al., 2024 [40]	Planned anthracycline (multicenter; *N* = 1011)	Pre-treatment 12-lead ECG (clinical)	Transfer-learned AI model (from ECG→LVEF estimation to CTRCD risk)	Cardiotoxicity within 2 years (time-to-event)	Time-dependent AUC ≈ 0.78; HR ~ 3.3 for AI high-risk group	Baseline risk stratification to guide monitoring intensity and preventive strategies before therapy begins	Endpoint heterogeneity across systems; not continuous monitoring; wearable generalization unproven; prospective outcome benefit not demonstrated
Oikonomou et al., 2025 [31]	Anthracycline or trastuzumab (retrospective; large dataset)	Pre-treatment ECG (clinical)	AI-enhanced ECG risk score	Any CTRCD and severe LV dysfunction (e.g., EF < 40%)	HR ~ 3.35 for any CTRCD; HR ~ 13.5 for severe dysfunction (risk stratification)	Identifies highly vulnerable subgroup pre-treatment, potentially enabling targeted prevention and surveillance	Retrospective; performance metrics (e.g., AUC) and prospective workflow impact need confirmation; wearable translation not established
Luna-Alcalá et al., 2024 [28]	Breast cancer; anthracycline ± trastuzumab (*N* = 50)	Lab-based ECG-derived HRV (non-wearable; baseline + 3 months)	HRV change metrics; statistical thresholding	Early cardiotoxicity (as defined in study)	Sens 75%; Spec 69% (threshold-based)	Supports autonomic digital biomarkers: HRV “reactivity/adaptability” may flag risk while patients remain asymptomatic	Small cohort; controlled setting, not continuous real-world wearables; confounding (anemia/infection/deconditioning) must be addressed; needs prospective wearable validation
Gulati et al., 2017 [24]	HER2 + patients receiving trastuzumab (observational)	ECG-derived HRV metric (clinical setting)	HRV metric (e.g., deceleration capacity)	CTRCD / LV dysfunction during therapy	Earlier signal than LVEF changes (qualitative in this review)	Suggests trastuzumab cardiotoxicity may have detectable early autonomic signatures	Metric availability varies; not wearable/continuous; thresholds not standardized; generalizability uncertain
WATCH Study (ongoing) [41]	Lymphoma/sarcoma survivors (prospective trial)	Mobile AI-ECG + smartwatch telemetry (HR/rhythm ± activity)	AI-ECG + multi-sensor integration (trial evaluation)	Asymptomatic LV dysfunction and survivorship endpoints	Ongoing (results pending)	Directly tests multi-sensor, real-world feasibility; may generate implementation-grade evidence	Evidence pending; trial outcomes needed; workflow, burden, and equity/fairness impacts must be quantified

Representative studies spanning cardio-oncology cardiotoxicity risk prediction/detection using ECG/HRV-based signals and AI/analytics. “Clinical utility” highlights how each approach could fit into a wearable-AI clinical pipeline; “Key limitations/validation” clarifies evidence maturity and generalizability

*Abbreviations: AI* artificial intelligence, *AUC* area under the receiver operating characteristic curve, *CTRCD* cancer therapy–related cardiac dysfunction, *ECG* electrocardiogram, *EF* ejection fraction, *HF* heart failure, *HR* hazard ratio, *HRV* heart rate variability, *LVEF* left ventricular ejection fraction, *MLP* multilayer perceptron, *N* sample size, *PPG* photoplethysmography, *PROs* patient-reported outcomes, *QT* QT interval

### Anthracycline-induced cardiotoxicity cohorts

Anthracyclines (like doxorubicin and epirubicin) are among the most studied drugs for cardiotoxicity, given their well-known association with dose-dependent cardiac dysfunction. Several studies have focused on breast cancer patients receiving anthracycline chemotherapy, as this group is sizable and at high risk. 

One notable prospective study by Chang et al. (2022) followed breast cancer patients through anthracycline therapy and attempted to predict cardiotoxicity (defined by LVEF drop or heart failure symptoms) using AI models [32]. Although patients did not wear advanced sensors continuously, the study collected a rich set of clinical data including serial echocardiograms and biomarkers. They applied machine-learning classifiers (logistic regression, random forest, k-nearest neighbors, gradient boosting, and a multilayer perceptron neural network) to baseline and early-treatment data to identify patients who would later develop cardiotoxicity. The best model achieved a modest AUC of 0.66 for predicting cardiotoxicity, with high sensitivity (~ 86%) but lower specificity (~ 53%), indicating many false positives. However, this performance profile with high sensitivity and modest specificity, rather than being solely a limitation, illuminates the model’s pragmatic value as a clinical triage tool. In a practical workflow, such a model functions as an early-warning system designed to ‘cast a wide net,’ ensuring that the vast majority of patients who might develop cardiotoxicity are identified for intensified surveillance (e.g., more frequent echocardiograms or biomarker tests). Consequently, a large cohort of patients who are truly at low risk could potentially be spared frequent, costly, and anxiety-provoking evaluations, pending prospective validation. This risk-stratification strategy enables the optimized allocation of finite healthcare resources, focusing clinical vigilance on those most in need, and thus represents a viable pathway for the real-world clinical integration of AI-driven monitoring. Notably, this study did not incorporate wearable data; it illustrates the baseline difficulty of prediction which continuous data from wearables might improve upon.

In contrast, a single-center study by Jacobs et al. (2024) took a different approach, using AI analysis of standard 12-lead ECGs to screen for cardiotoxicity *after* anthracycline chemotherapy [39]. They trained a deep learning model (a form of convolutional neural network) on ECG recordings to detect reduced LVEF. The algorithm was applied to nearly 1,000 breast cancer patients post-anthracycline, and it predicted newly abnormal LVEF (< 50%) with an impressive AUC of ~ 0.93. This high accuracy suggests that even without wearables per se, AI applied to readily available signals like ECG can greatly aid cardiotoxicity screening. It’s plausible that continuous wearable ECG monitoring could feed similar algorithms in real-time. Jacobs et al.’s work demonstrates that subtle ECG changes can presage a drop in ejection fraction, validating the concept of noninvasive early warning. 

Another advanced study, by Yagi et al. (2024), leveraged a deep neural network to predict chemotherapy-induced cardiotoxicity *before* therapy initiation using pre-treatment ECG data [40]. This was a multi-center study of over 1,000 anthracycline-treated patients, employing an AI algorithm initially trained to estimate LVEF from ECGs. Researchers adapted that model via transfer learning to specifically predict cardiotoxicity occurrence within two years post-chemotherapy. The model’s time-dependent AUC was 0.78, compared to 0.74 using traditional risk factors alone, a statistically significant improvement. Moreover, patients flagged as high-risk by the AI model had a hazard ratio of over 3 for developing CTRCD. This underscores that AI can stratify patients at baseline, identifying those who might benefit from closer monitoring or prophylactic cardio-protection.

Moving to HRV-focused research: a small but illuminating study by Luna-Alcalá S, et al. (2024) in Mexico examined autonomic changes in 50 breast cancer patients during anthracycline ± trastuzumab therapy, aiming to see if heart rate variability (HRV) could predict early cardiotoxicity [28]. They measured HRV under controlled conditions at baseline and 3 months after chemo. Results showed that while absolute HRV indices did not differ significantly, the *change* in HRV under certain conditions was predictive. In particular, a smaller drop in HRV during deep breathing was associated with subsequent cardiotoxicity. A specific threshold yielded ~ 75% sensitivity and ~ 69% specificity for early cardiotoxicity detection. This suggests reduced autonomic adaptability might be a warning sign. Although this study took HRV readings in a lab rather than via a continuous wearable, its findings strongly support using HRV metrics in monitoring, as these patients were asymptomatic at 3 months when the difference was apparent.

### HER2-targeted therapy (trastuzumab) cardiotoxicity

HER2-targeted therapies, especially trastuzumab, cause a form of cardiotoxicity that is often reversible but still requires close monitoring. While anthracycline damage is often permanent, trastuzumab seems to cause myocardial stunning that can recover if therapy is interrupted.

The previously mentioned HRV studies included patients on combined regimens. Gulati et al. (2017) observed that among HER2 + patients on trastuzumab, those who developed cardiotoxicity had greater drops in a specific HRV metric (deceleration capacity), which could predict dysfunction earlier than LVEF changes [24]. This implies that even for trastuzumab’s often transient cardiotoxicity, early autonomic changes occur and are detectable.

A retrospective analysis by Oikonomou et al. (2025) examined a large dataset of patients who received either anthracycline or trastuzumab, using an AI model applied to pre-treatment ECGs for risk stratification [31]. Their AI-enhanced ECG risk score identified a subset of patients with a dramatically higher incidence of cardiac dysfunction on follow-up, with hazard ratios of ~ 3.35 for any CTRCD and ~ 13.5 for severe LV dysfunction (EF < 40%) if the AI classified them as high-risk. This included trastuzumab patients, suggesting that baseline ECG features can stratify who is vulnerable to trastuzumab’s cardiotoxic effects too.

Specific studies focusing on wearables in trastuzumab-only populations are scant so far. One could hypothesize that trastuzumab cardiotoxicity might manifest as milder changes given its typically reversible nature—e.g., a slight persistent tachycardia or small decreases in exercise capacity could be early clues. A Canadian pilot study named REMOTHEART was testing remote monitoring including blood pressure and symptom reporting for HER2-positive patients, but results are pending.

Notably, the concept echoes the SUCCOUR trial, where myocardial strain imaging was used to guide cardioprotective interventions during anthracycline therapy [42]. Similarly, wearable-derived metrics and AI-enhanced tools may offer individualized guidance for managing trastuzumab-related cardiotoxicity in the future.

### Other agents and combination therapies

Beyond the classic agents, newer cancer therapies also have cardiovascular side effects that might benefit from monitoring. Immune checkpoint inhibitors (ICIs) can cause an immune-mediated myocarditis, which is infrequent (around 1–2%) but often acute and severe. One can speculate that a wearable ECG could catch episodes of non-sustained ventricular tachycardia or new conduction abnormalities that herald myocarditis. While no dedicated studies have reported this yet, case reports exist where patients with ICI myocarditis had warning signs that, had they been monitored, might have allowed earlier intervention [43].

Tyrosine kinase inhibitors (TKIs) can cause hypertension and QT prolongation. For these, remote blood pressure monitoring via wearables or home devices is very relevant. Similarly, vascular endothelial growth factor (VEGF) inhibitors are well-known for inducing or exacerbating hypertension, representing another major drug class where remote blood pressure surveillance could prove invaluable for timely management. An AI approach could integrate blood pressure trends with heart rate and weight to detect early development of hypertension or volume retention. Some TKIs also cause arrhythmias, and a wearable device could periodically check the QT interval via a single-lead ECG algorithm [10, 44]. 

Many patients receive multiple cardiotoxic therapies and are at especially high risk. One small observational study of lymphoma survivors showed an AI-enhanced ECG algorithm on a smartphone was able to detect reduced LV function after anthracycline therapy [45]. A key ongoing trial, the WATCH study, is testing a combination of AI ECG and smartwatch data to surveil survivors of lymphoma and sarcoma who had cardiotoxic therapies [41]. Preliminary reports suggest high accuracy of a mobile AI-ECG in detecting asymptomatic LV dysfunction, and they are examining whether a smartwatch’s alerts add further value.

### Endpoints and performance metrics

The studies to date use a variety of endpoints to define “cardiotoxicity,” which is important when comparing results. Common endpoints include: a specific LVEF drop (e.g., ≥ 10% drop to < 50% LVEF), new heart failure symptoms or diagnosis (MACE), or biomarker elevation.

The choice of endpoint affects model performance. Predicting a hard endpoint like heart failure hospitalization might be easier in some cases but is rarer, whereas predicting any EF drop is sensitive but could include minor, reversible changes. The typical performance metrics reported include area under the ROC curve (AUC), sensitivity, specificity, and sometimes the lead time (how far in advance of conventional detection the algorithm could give an alert). Some studies also report negative predictive value, which is clinically useful to rule out risk.

The acceptable balance between sensitivity and specificity depends on the clinical context. In monitoring, higher sensitivity is often preferred to ensure most issues are caught, even if it leads to more false positives that require confirmatory testing like an echocardiogram. For instance, the Chang et al. study prioritized sensitivity (~ 86%) over specificity (~ 53%), meaning their model flagged many patients for closer observation.

Finally, one must note the critical limitation of external validation: few of these models have been validated outside their original study population yet. Broader validation is a key step that is still largely lacking and is necessary before these models can be considered for widespread clinical use.

### Synthesis of evidence: model performance, clinical insights, and current boundaries

Across representative studies, reported discrimination for identifying patients with or at risk for CTRCD-related endpoints ranges from modest to high, depending on the signal modality (wearable telemetry vs. clinical ECG), endpoint definition, and cohort size. Importantly, most published results should be interpreted as risk stratification or screening augmentation, not as stand-alone diagnostic tests. In early-stage cohorts, models that prioritize sensitivity may be most clinically useful as triage tools that trigger confirmatory echocardiography/biomarker testing [32], while models with higher specificity may reduce unnecessary evaluations but risk missed events if used without safeguards.

Three clinical insights recur across the current literature: (1) baseline or early-treatment physiologic drift (e.g., rising resting HR, falling parasympathetic HRV indices, increasing rhythm irregularity burden) may precede conventional detection [24, 28]; (2) multimodal context (activity/sleep) can reduce misinterpretation of heart rate changes driven by non-cardiac factors [22]; and (3) interpretability is likely a prerequisite for clinician trust, particularly when alerts may affect cancer therapy decisions [31]. 

The major boundary is evidence maturity: many studies are single-center, event-limited, or use heterogeneous endpoints, and few have robust external validation. Therefore, the field should be described as promising but not yet practice-changing until multicenter validation and randomized trials demonstrate improved clinical outcomes and acceptable workflow burden [41]. Table 4 maps representative studies against the proposed end-to-end pipeline, highlighting current gaps in workflow integration and prospective validation.

**Table 4:** How existing studies align with an end-to-end wearable-AI “data → decision” clinical pipeline

Study	Continuous/near-continuous monitoring	Multimodal context (beyond single signal)	Personal baseline calibration / drift framing	QC & missingness strategy described	Interpretability / explanation layer	External validation / multicenter evidence	Prespecified clinical escalation pathway	RCT / pragmatic trial evidence
Chang et al., 2022 [32]	—	◐ (clinical + echo/biomarkers)	◐ (baseline/early-treatment predictors, not individualized drift)	—	—	—	—	—
Jacobs et al., 2024 [39]	—	—	—	—	—	—	—	—
Yagi et al., 2024 [40]	—	—	—	—	—	✓ (multicenter cohort)	—	—
Oikonomou et al., 2025 [31]	—	—	—	—	—	—	—	—
Luna-Alcalá et al., 2024 [28]	— (two time points only)	—	◐ (change-from-baseline HRV in controlled setting)	—	—	—	—	—
Gulati et al., 2017 [24]	—	—	◐ (HRV metric change concept)	—	—	—	—	—
WATCH Study (ongoing) [41]	✓	✓ (AI-ECG + smartwatch telemetry)	◐ (likely feasible; prespecification pending)	◐ (expected in trial, details pending)	◐ (depends on trial reporting)	◐ (prospective; broader validation pending)	◐ (trial protocol-driven)	◐ (prospective trial; RCT status not established here)

Mapping of representative studies to key translational components required for safe clinical deployment. ✓ = explicitly included/described; ◐ = partially/implicitly addressed; — = not described or not included based on information summarized in this review

*Abbreviations:AI* artificial intelligence, *ECG* electrocardiogram, *Echo* echocardiography, *HF* heart failure, *HRV* heart rate variability, *LVEF* left ventricular ejection fraction, *MLP* multilayer perceptron, *PROs* patient-reported outcomes, *QC* quality control, *RCT* randomized controlled trial

## Discussion: from subclinical digital biomarkers to clinical action

Current evidence suggests that autonomic and functional changes measured by wearables may capture an early trajectory of cardiac stress during cardiotoxic therapy—often before an overt LVEF decline is documented. A plausible clinical arc is that subtle autonomic imbalance (sympathetic predominance) and impaired physiologic recovery manifest as a sustained increase in resting or nocturnal heart rate and a decline in parasympathetic HRV indices [24, 28]. Over time, these changes may coincide with reduced activity tolerance and sleep disruption, eventually progressing to recognizable symptoms such as exertional dyspnea, edema, or tachyarrhythmias [25]. Importantly, these signals are not specific to CTRCD; they can also reflect anemia, infection, pain, deconditioning, or cancer-related cachexia [2]. This reinforces the need for multimodal interpretation and confirmatory testing pathways.

### Do actionable thresholds exist? A pragmatic operational trigger framework

At present, there are no universally validated wearable thresholds (for HR or HRV) that define “early cardiotoxicity” across cancer populations and therapies. Inter-individual variability is large, and device algorithms differ. Therefore, the most defensible near-term approach is an individualized, baseline-relative trigger that is explicitly designed to prompt evaluation rather than to diagnose CTRCD. Examples of operational triggers that could be prospectively tested include: (1) sustained resting or nocturnal HR elevation relative to baseline for ≥ 7–14 days, (2) sustained decline in parasympathetic HRV indices (e.g., RMSSD) relative to baseline, particularly when paired with rising nighttime HR, (3) concurrent functional deterioration (step count or activity minutes) and symptom escalation, and (4) rhythm alerts (new atrial fibrillation/flutter, increasing ectopy burden) in relevant therapeutic contexts [16]. These triggers should be treated as hypothesis-generating, and future trials must quantify their sensitivity, specificity, lead time, and downstream clinical impact.

### What success should look like in trials

For wearable-AI cardiotoxicity monitoring to justify routine adoption, it must demonstrate benefit beyond improved AUC. Key outcomes include earlier confirmatory testing, prevention of severe CTRCD, improved tolerance/completion of cancer therapy, patient-centered outcomes (symptom burden, quality of life), reduced unplanned admissions, and acceptable clinician workload. This requires prospective, multicenter study designs with prespecified escalation pathways and rigorous monitoring for unintended harm (alert fatigue, anxiety, inequity) [41].

### Challenges & limitations

While the prospect of wearable AI-driven monitoring for cardiotoxicity is promising, significant translational barriers remain. As summarized in Table 4, relatively few studies report the full set of translational components required for routine clinical adoption (e.g., external validation, prespecified escalation pathways, or pragmatic trial evidence).

1. Small, Heterogeneous Cohorts & Lack of External Validation

Many current studies are limited by small sample sizes (often < 200 patients) and single-center designs, which raises concerns about the overfitting of AI models. Patient populations are frequently heterogeneous, with varying cancer types and treatment regimens, meaning a model trained on one specific cohort may not generalize to another. A critical gap is the lack of robust external validation; few models have been tested on independent datasets from different health systems. Furthermore, moderate-to-severe cardiotoxicity events are relatively infrequent, creating a “class imbalance” problem that makes training robust classifiers difficult without large-scale data pooling.

2. Data Privacy and Security

Continuous monitoring generates vast amounts of sensitive physiological data, raising significant privacy concerns under regulations like GDPR and HIPAA. Ensuring robust cybersecurity is paramount to protect data stored on cloud servers. Smart-healthcare deployment frameworks emphasize that interoperability, cybersecurity, and governance are core determinants of scalable adoption [15]. Secure data pipelines must be developed, and strategies like on-device processing (edge computing) are increasingly needed to minimize the privacy risks associated with transmitting raw telemetry.

3. Device Adherence and Data Quality

The utility of any wearable-based system depends on sustained patient adherence, which often wanes over time. In the context of chemotherapy, side effects like skin irritation or general patient burden may reduce compliance [46]. Data quality is intrinsically linked to adherence and is susceptible to motion artifacts. A significant portion of collected data may be unusable, necessitating algorithms that are robust to noise and missingness. Moreover, socioeconomic barriers to device access and digital literacy gaps, particularly in older populations, must be addressed to ensure equitable deployment.

4. Model Generalizability & Algorithmic Bias

AI models can inherit and amplify biases present in their training data. A critical concern in cardio-oncology is algorithmic bias, particularly regarding sensor accuracy. For instance, it is a known limitation that PPG sensors can be less accurate on darker skin tones due to melanin absorption properties, potentially leading to health disparities if models are not calibrated across diverse populations [47, 48]. Ensuring equity requires training models on inclusive datasets and rigorously auditing performance across demographic subgroups before deployment. Additionally, federated learning is a promising approach to train models across multiple institutions without sharing raw patient data, thereby improving generalizability while preserving privacy [49].

5. Clinical Workflow Integration and Alert Fatigue

A major practical barrier is integrating continuous data into clinical workflows without overwhelming healthcare providers. Excessive false-positive notifications can lead to alert fatigue, causing clinicians to desensitize to warnings. Successful implementation requires clear, human-in-the-loop protocols where AI outputs are triaged by specialized staff (e.g., nurses) before escalating to oncologists. Furthermore, the black-box nature of many deep learning models remains a hurdle; without interpretability, clinicians may hesitate to act on an AI risk score, reinforcing the need for explainable AI (XAI) features that link predictions to physiological logic.

### Future directions & recommendations

To realize the full potential of this field, several key developments are recommended.

1. Harmonized Data Standards & Shared Repositories

Establishing common data standards for wearable data in cardio-oncology is essential, including standardized data formats and consistent definitions for clinical events. This would enable the creation of large, multi-center public data repositories, similar to resources like PhysioNet [50]. Pooling anonymized data would dramatically accelerate AI model training and validation.

2. Prospective, Multicenter Validation and Randomized Trials

Beyond retrospective external validation, prospective multicenter studies and robust randomized controlled trials are now essential to generate practice-grade evidence. Pragmatic RCTs (or cluster-randomized implementation trials) should compare wearable-AI–supported surveillance versus standard care in high-risk populations, using prespecified escalation pathways and clinically meaningful endpoints (e.g., incidence of moderate/severe CTRCD, heart-failure hospitalization, therapy interruption, and patient-reported outcomes) [41]. Trials should also evaluate health economics, equity (subgroup performance), and clinician workload. Without this level of evidence, wearable-AI systems should be framed as investigational decision-support tools rather than routine standards of care.

3. Integration into Tele-Cardio-Oncology Care Platforms

The evolution of cardio-oncology care depends on integrated digital health platforms that unify oncology and cardiology care. Wearable data should flow seamlessly into electronic health records (EHRs) or specialized cardio-oncology dashboards. This would enable a tele-cardio-oncology model where AI-generated alerts trigger timely telehealth consultations and remote interventions, building on the momentum gained by remote care during the COVID-19 pandemic [51].

4. Advances in Technology and Analytic Methods

On the technology side, future wearables may incorporate novel sensors, such as those for tracking biochemical markers. On the analytics side, federated learning will be crucial for training on diverse, private datasets. Furthermore, multi-modal AI that fuses wearable data with information from EHRs, genomics, and imaging holds the potential to create far more accurate and personalized prediction models [22].

5. Development of Novel, Digital Composite Endpoints

To advance the standard of care from reactive to proactive care, future research must transcend the reliance on traditional, lagging endpoints such as LVEF decline. There is a compelling need for the development and validation of a novel, digital composite endpoint that provides a holistic, real-time measure of a patient’s well-being. Such an endpoint should integrate: (1) continuous physiological metrics from wearables (e.g., a sustained decrease in HRV or increase in resting heart rate); (2) patient-reported outcomes (PROs) captured via digital platforms (e.g., escalating fatigue or dyspnea); and (3) objective behavioral data (e.g., a significant decline in daily step count or functional activity). Establishing this dynamic, multi-modal endpoint in prospective trials will be critical to redefining therapeutic success—moving the goal from simply identifying established cardiac damage to proactively maintaining the patient’s overall physiological resilience throughout their cancer journey.

6. Regulatory Pathways, Reimbursement, and Ethics

Clear regulatory pathways, such as the FDA’s framework for Software as a Medical Device (SaMD), are needed. Developers must engage with regulators early and follow rigorous reporting guidelines like CONSORT-AI to build trust [52]. Reimbursement models must also evolve to support these new care models. Ethically, frameworks must be established to guide clinical decision-making based on AI-generated risk scores, ensuring that patient preferences are central to the process through shared decision-making [53].

## Conclusions

Chemotherapy-induced cardiotoxicity requires innovative, interdisciplinary solutions. Wearable sensors and AI-based early warning systems offer significant promise to enhance cardio-oncology surveillance. By enabling continuous, real-time monitoring, this approach can detect the subtle, early signs of cardiac stress long before they are apparent with traditional methods.

While substantial challenges remain, from technical hurdles of data quality to practical issues of clinical integration, the field is rapidly advancing. Achieving this vision requires not only rigorous validation but also a fundamental re-evaluation of our clinical endpoints. By developing and adopting digital composite endpoints that fuse physiological data with patient-reported outcomes and activity levels, we can redefine success. The ultimate promise of integrating wearable technology and AI is therefore not merely to predict injury, but to evolve cardiotoxicity surveillance into a proactive, continuous process of maintaining cardiovascular wellness. This proactive approach may be instrumental in allowing patients to complete curative cancer therapies more safely, ensuring that survivorship is defined not just by the absence of cancer, but by a sustained quality of life and long-term cardiovascular health.

## Data Availability

No datasets were generated or analysed during the current study.

## Abbreviations

AI: Artificial Intelligence
CIC: Chemotherapy-induced cardiotoxicity
CTRCD: Cancer therapy–related cardiac dysfunction
ECG: Electrocardiogram
HRV: Heart rate variability
LVEF: Left ventricular ejection fraction
LSTM: Long Short-Term Memory
PPG: Photoplethysmography
RNN: Recurrent Neural Network
XAI: Explainable Artificial Intelligence
